# Supplementary material for: Anti-Invasive and Anti-Migratory Effects of Ononin on Human Osteosarcoma Cells by Limiting the MMP2/9 and EGFR-Erk1/2 Pathway
Source: Cancers (Basel). 2023 Jan 26;15(3):758. doi: 10.3390/cancers15030758 (PMC9913877; doi:10.3390/cancers15030758)

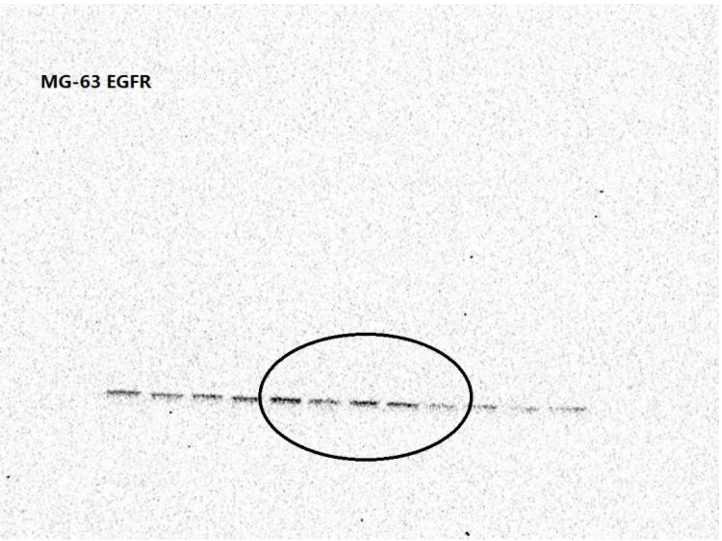
MG-63 EGFR

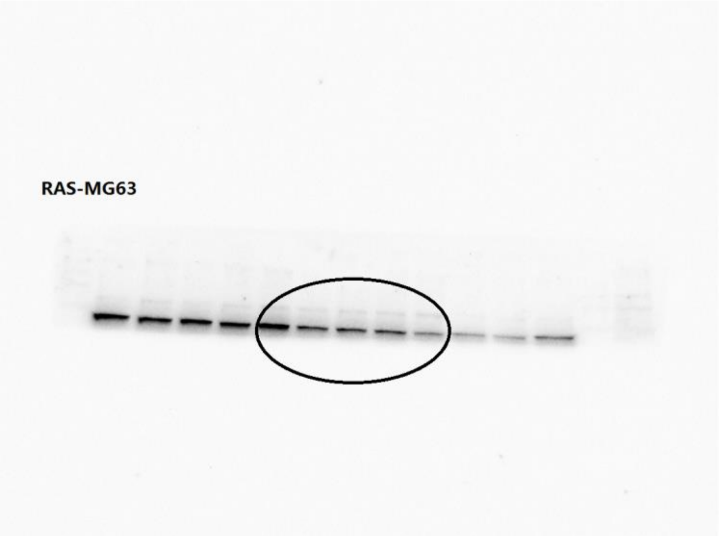
RAS-MG63

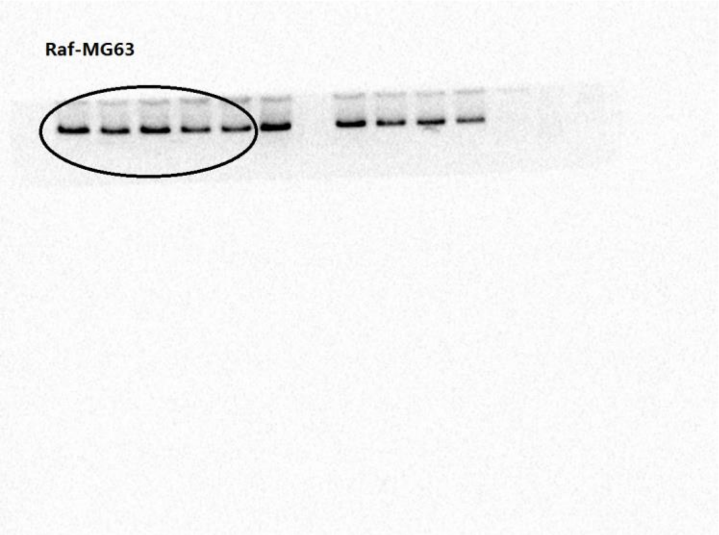
Raf-MG63

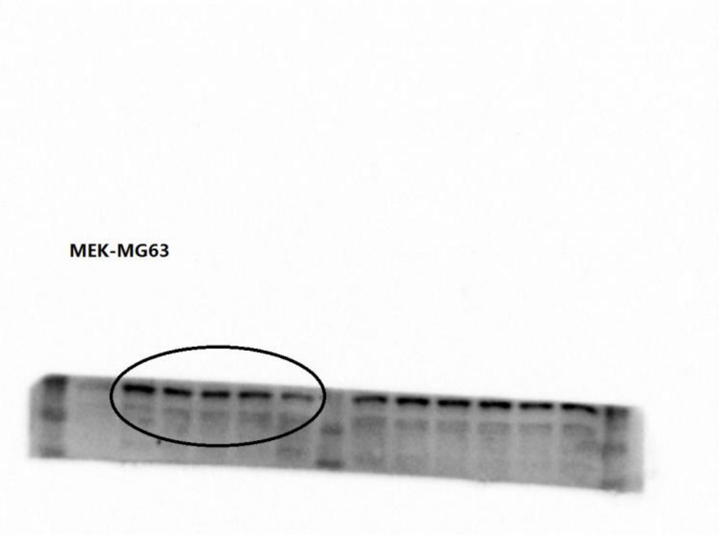
MEK-MG63

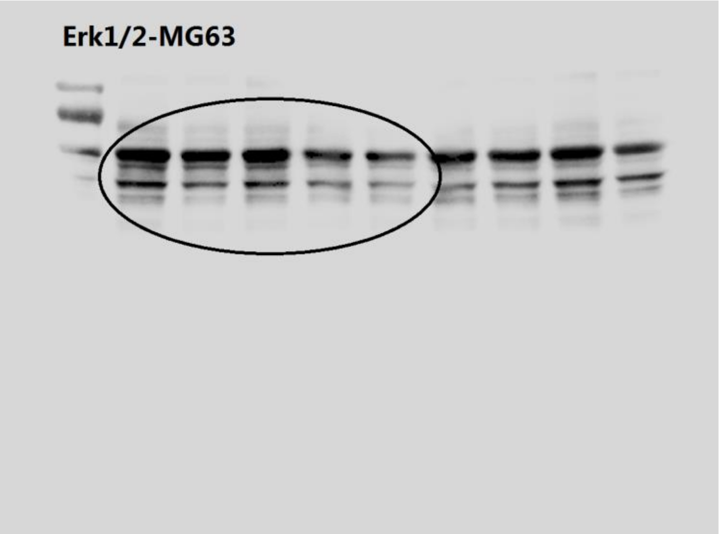
Erk1/2-MG63

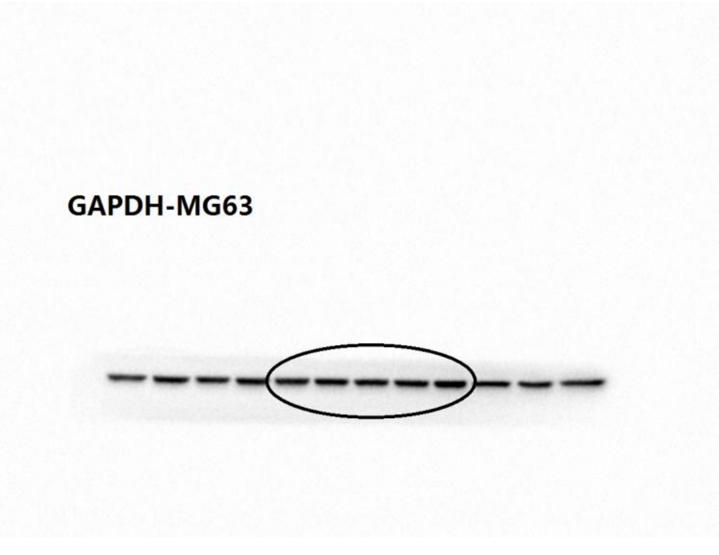
GAPDH-MG63

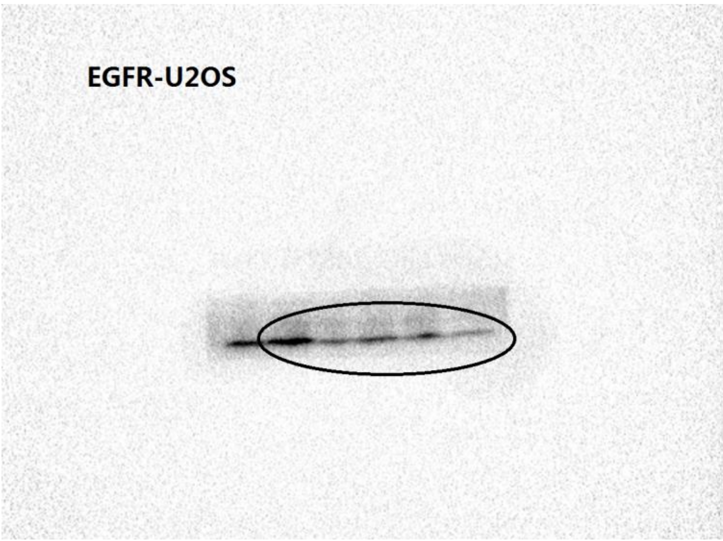
EGFR-U2OS

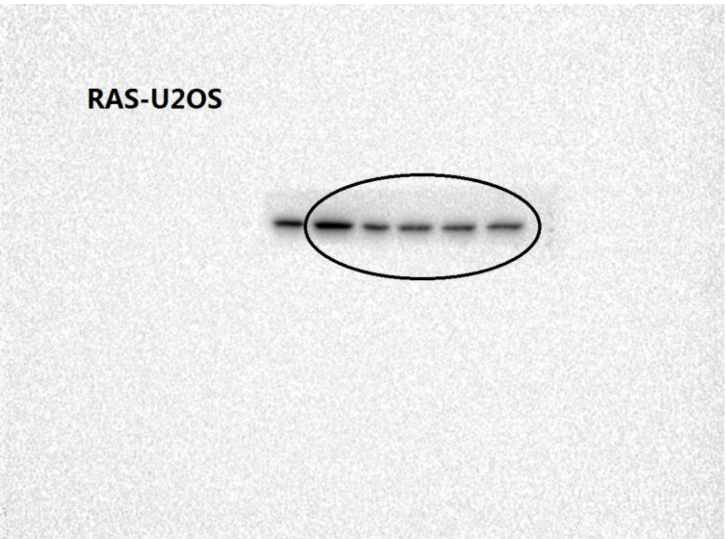
RAS-U2OS

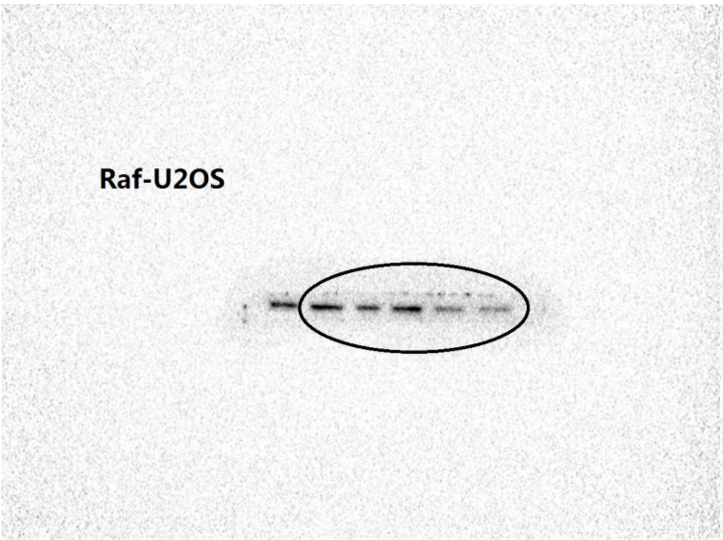
Raf-U2OS

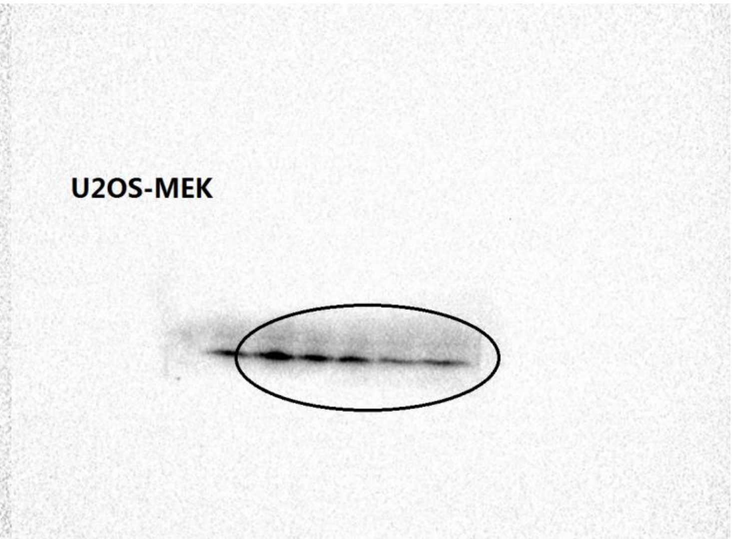
U2OS-MEK

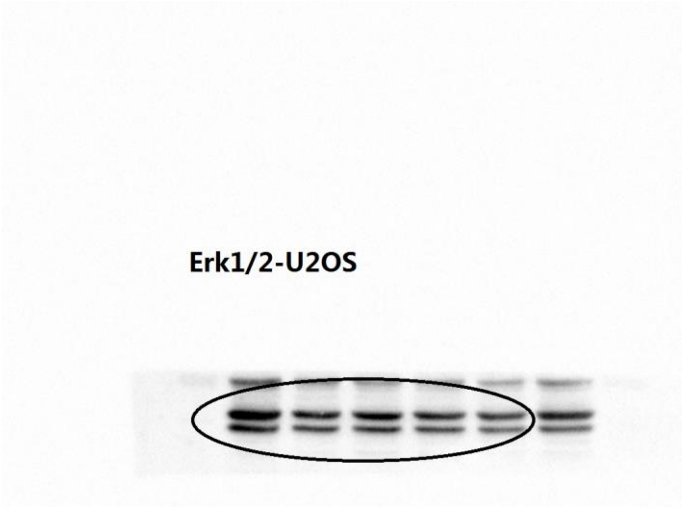
Erk1/2-U2OS

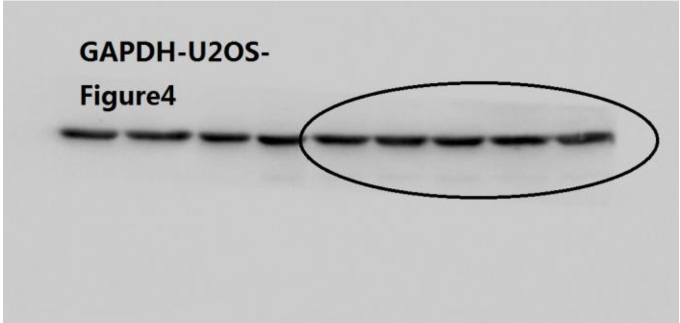
GAPDH-U2OS-
Figure4

MMP-2 MG63

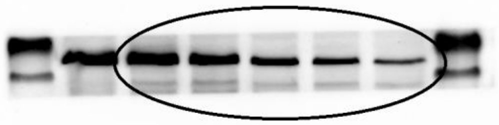

MMP-9 MG63

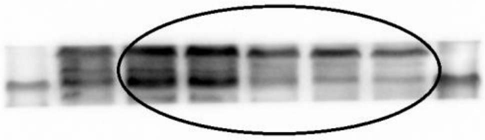

Vimentin MG63

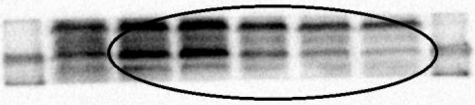

Figure 5 MG-63 loading control

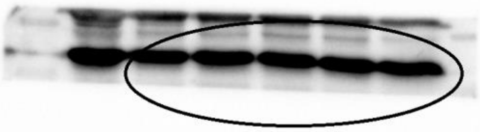

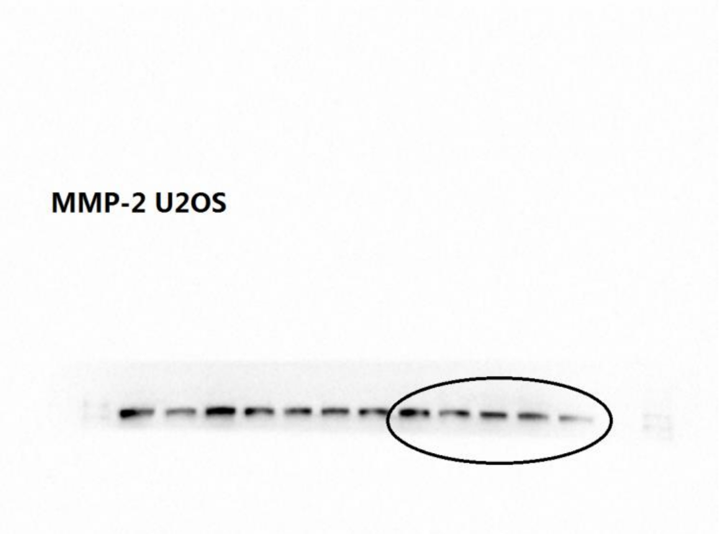
MMP-2 U2OS

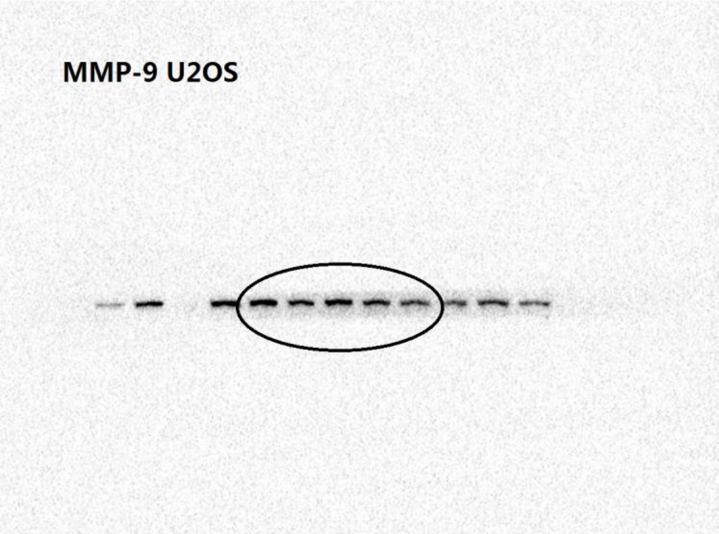
MMP-9 U2OS

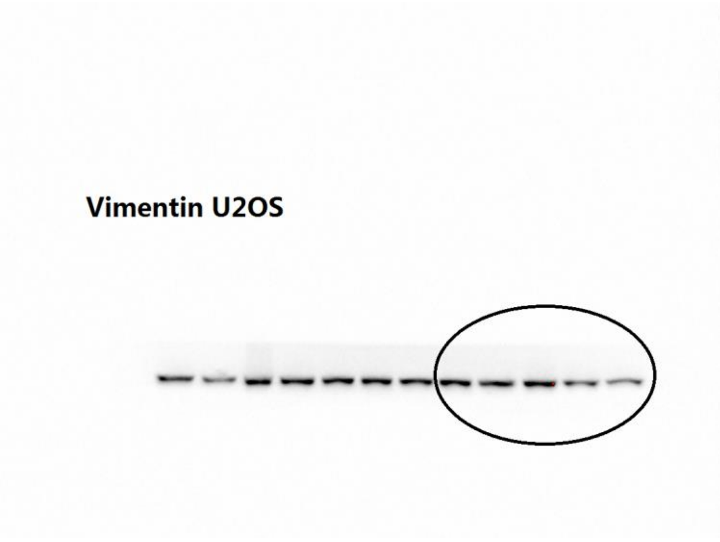
Vimentin U2OS

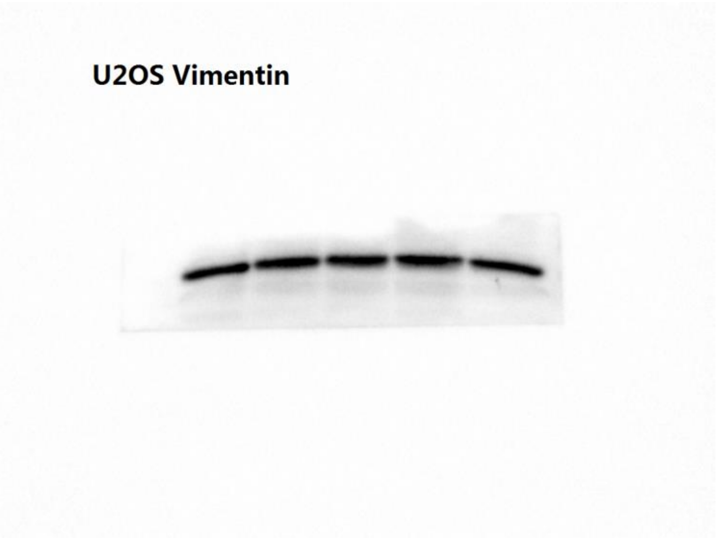
U2OS Vimentin

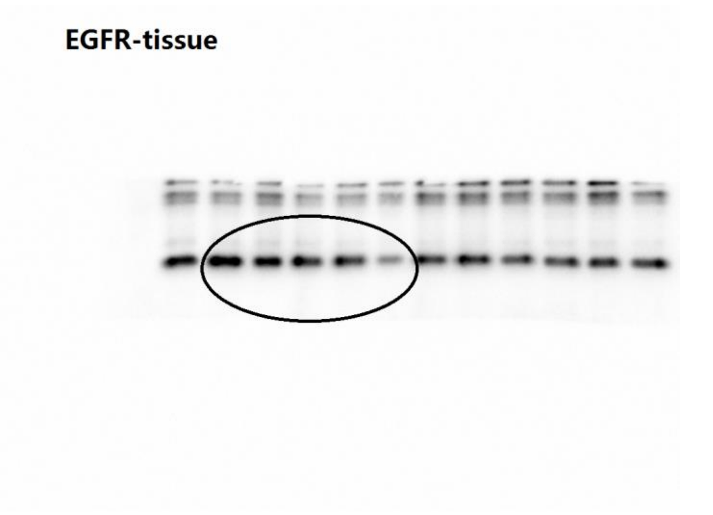
EGFR-tissue

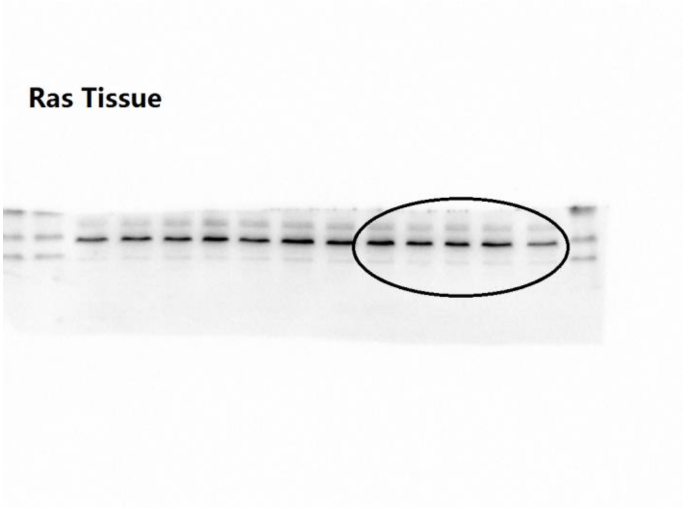
Ras Tissue

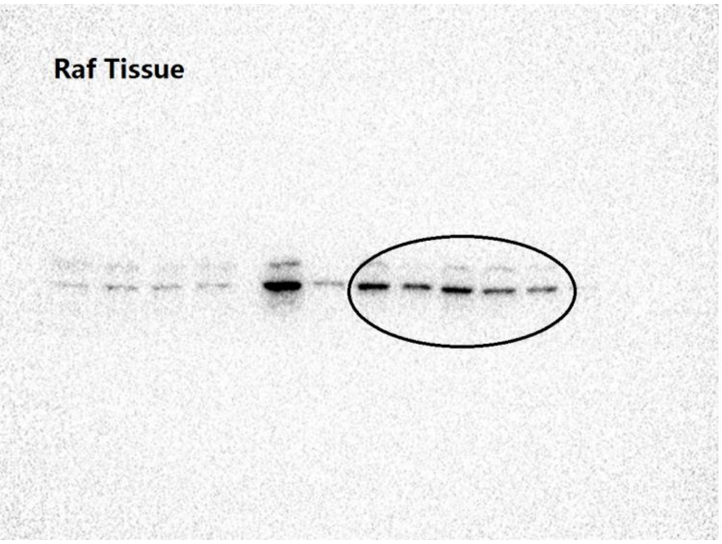
Raf Tissue

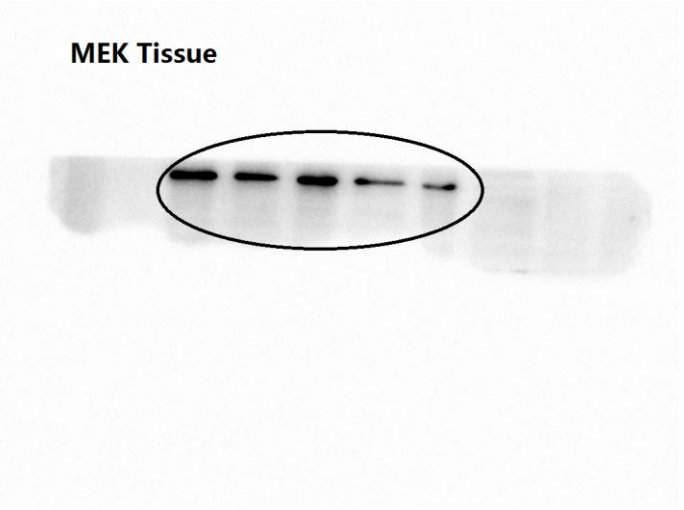
MEK Tissue

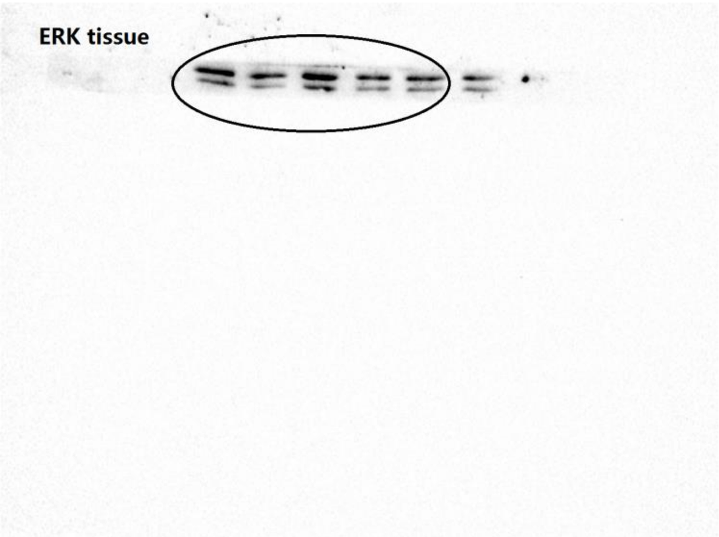
ERK tissue

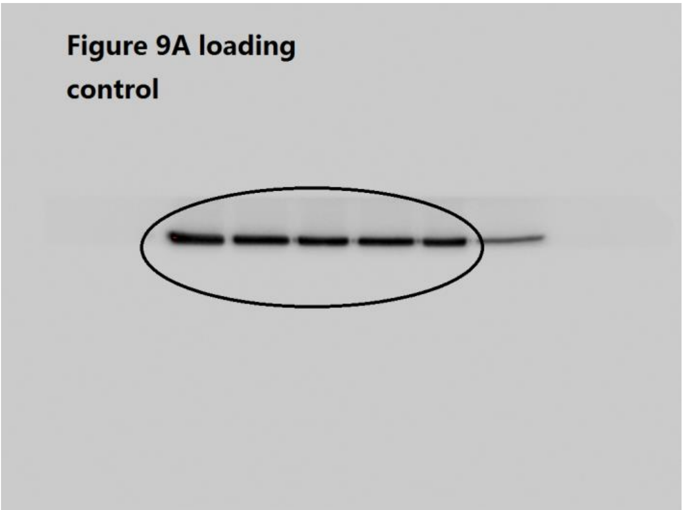
Figure 9A loading
control

MMP-2 tissue

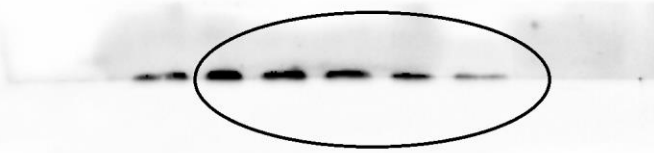

MMP-9
tissue

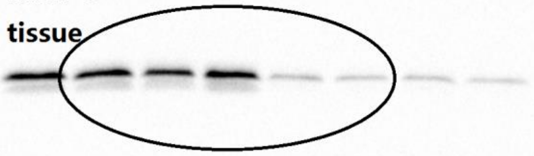

Vimentin Tissue

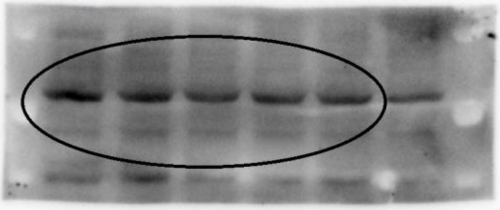

Figure 9B GAPDH

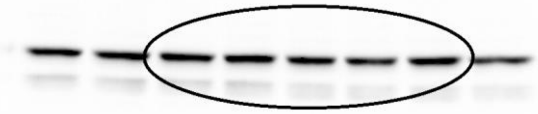

Supplement: Supplementary file 1 [file cancers-15-00758-s001.zip › cancers-2148129-supplementary.pdf]
